# Bloody Tumour of Bone

**Published:** 1829-07-01

**Authors:** 


XXIV.

Bloody Tumour of Bone.

The first case of this curious disease will be found, we believe, in the works of Mr. Pott, who observed it in the calf of the leg, and imputed it to a diseased condition and rupture of the posterior tibial artery. In this pathological supposition it seems, from the cases which have since been detailed by other authors, by Scarpa, Mr. Pearson, Mr. Else, Mr. Freer of Birmingham, Mr. Hodgson, and still more lately by M.M. Lallemand and Breschet,* that Mr. Pott was wrong. It appears that the disease resembles closely the aneurism by anastomosis, being seated in the smaller arteries not of the soft parts, but of the bones themselves, and especially the tibia and fibula. M. Breschet strongly recommends the application of a ligature to the femoral artery for the cure of this affection in its early stage, but knowing what we know respecting the failure of the ordinary operation for aneurism in cases of aneurism by anastomosis elsewhere, we are not only sceptical of the value of M. Breschet's proposition, but even dissent from it in toto. The profession are beginning to discover, that they have run the Hunterian operation for aneurism a little too hard, and applied it to cases to which it was in every point of view inapplicable. In the work of Mr. Bell on Diseases of the Bones, so fully noticed in the Review department of the last number, is detailed an interesting case of the affection in question. Every contribution to the stock of facts on a subject of which we know so little is a valuable boon to the profession. As the case is short, we give it in the author's words.

"The patient was a lady about sixty years of age, who had suffered from what she imagined to be rheumatism of the upper arm of the right side, at least, the arm was painful on being handled, and a feeling of aching was referred to the bone. In the course of several months after the first symptoms of the bone being affected had appeared, the upper arm began to swell, and the enlargement went on progressively increasing, until the middle portion of the diseased humerus measured more than double the circumference of the sound one. She suffered considerable uneasiness in the part, and the pain extended downwards to the points of the fingers. The surface of the tumour was not discoloured, but several large and swollen veins coursed over it. Before applying to my father she had consulted a bone-setter, who, according to the principles of his craft, recommended frictions and shampooing. This practice, the patient affirmed, had the effect of exasperating the disease, and of inducing intolerable pain.

"Although the real nature of the complaint was not suspected, still as it was on the increase, and presented some of those characters which are met with in osteo-sarcoma in its middle stages, and in spina ventosa in its more advanced, it was deemed proper, by the several surgeons assembled in consultation, among whom were Professor Russell, Dr. Abercrombie, and Mr. Craig of Ratho, to amputate the limb. My father accordingly removed the arm from the socket of the shoulder-joint; there was little bleeding at the time, although the arteries which supplied the upper part of the limb with blood, were unusually dilated. About six hours after the operation, however, secondary hæmorrhage occurred, and it was found necessary to secure three large vessels,

* A full account of the Mémoires of these gentlemen will be found in Vol. VII. New Series, of this Journal, to which we refer our readers for information respecting the disease.

which had not thrown out blood during the operation. This patient recovered in a short time from the operation, and *suffered no return of the complaint*, but died two years afterwards, from a sudden attack of pleuritis.

"In order to ascertain whether any connection existed between the great vessels of the limb and the tumour of the bone, I injected the arteries of the arm from the humeral artery, with a preparation of glue and vermilion. The injection ran kindly, and flowed through the minutest of the distal arterial ramifications.

"On removing the muscles, the tumour was found to be confined entirely to the bone. It was, when divested of the soft parts, nine inches in circumference, and six in length. The periosteum was entire, and somewhat thickened. On cutting into the tumour, the scalpel grated upon what seemed to be osseous depositions, which pervaded its tissue; but its general texture was of a fibrous character. A thin, though imperfect, shell of bone, which was internal to, but in immediate contact with, the periosteum, nearly coated the tumour. There was a large cavity in the centre of the tumour, lined by an organized membrane, into the vessels of which the size injection had flowed; and the cavity itself was filled, partly with fluid blood of a dark colour, partly with concentric coagula, and partly with hardened injection.

"From the pathological appearances presented in this preparation, I have no hesitation in esteeming it as a specimen of what M. Breschet has termed Aneurism of Bone; and several able anatomists to whom I have shewn it coincide in opinion with me."

An etching of the bone, shewing the tumour in situ, and its section, is appended, but little of course can be learnt from it. We cannot dismiss the case without again expressing our conviction, that the disease is not amenable to the ordinary operation for aneurism, and that whether in its earliest stages it be or be not remediable by nature or by art, that amputation is the only remedy when once it has acquired maturity and magnitude. It is not unlikely that cases of the disease have occurred to professional men, which have not met the eye of the public, but if any such there be, we trust that their possessors will enrich their brethren with the details.

